# Licochalcone A Suppresses Migration and Invasion of Human Hepatocellular Carcinoma Cells through Downregulation of MKK4/JNK via NF-κB Mediated Urokinase Plasminogen Activator Expression

**DOI:** 10.1371/journal.pone.0086537

**Published:** 2014-01-22

**Authors:** Jen-Pi Tsai, Pei-Ching Hsiao, Shun-Fa Yang, Shu-Ching Hsieh, Da-Tian Bau, Chu-Liang Ling, Chun-Li Pai, Yi-Hsien Hsieh

**Affiliations:** 1 Department of Nephrology, Buddhist Dalin Tzu Chi General Hospital, Chiayi, Taiwan; 2 Institute of Medicine, Chung Shan Medical University, Taichung, Taiwan; 3 School of Medicine, Chung Shan Medical University, Taichung, Taiwan; 4 Department of Internal Medicine, Chung Shan Medical University Hospital, Taichung, Taiwan; 5 Graduate Institute of Clinical Medical Science, China Medical University, Taichung, Taiwan; 6 Department of Biotechnology, Asia University, Taichung, Taiwan; 7 Institute of Biochemistry and Biotechnology, College of Medicine, Chung Shan Medical University, Taichung, Taiwan; 8 Department of Biochemistry, School of Medicine, Chung Shan Medical University, Taichung, Taiwan; 9 Department of Clinical Laboratory, Chung Shan Medical University Hospital, Taichung, Taiwan; The University of Hong Kong, China

## Abstract

Hepatocellular cell carcinoma (HCC) is one of the most commonly diagnosed cancers worldwide and in Taiwan. Chemoprevention of cancer with dietary bioactive compounds could potentially reverse, suppress, or prevent cancer progression. Licochalcone A (LicA) is a characteristic chalcone of licorice, which is the root of Glycyrrhiza inflate. It had been reported that LicA has anti-inflammatory, anti-microbial, and anti-tumor properties. However, the effects of LicA on the migration and invasion of human HCC cells have not yet been reported. In the present study, it was found that LicA inhibits the migratory and invasion ability of SK-Hep-1 and HA22T/VGH cells in a dose-dependent manner, as assessed by the cell migration and Matrigel cell invasion assay. Using casein zymography, Western blotting, reverse transcriptase polymerase chain reaction, and an immunofluorescence assay, it was found that LicA induces a dose-dependent inhibition of uPA activity and expression, as well as reduces mRNA levels in SK-Hep-1 and HA22T/VGH cells. LicA was also found to inhibit the expression of phosphor-JNK and phosphor-MKK4 in SK-Hep-1 cells. Furthermore, LicA significantly decreased uPA levels in SP600125-treated or si-MKK4-transfected cells alongside a marked reduction in cell migration and invasion, which supports the notion that an inhibition of MKK4/JNK results in anti-metastatic effects. Moreover, LicA inhibited the expression of nuclear NF-κB, as well as the binding ability of NF-κB to the uPA promoter. These findings further our understanding of the role of LicA in suppressing tumor metastasis and its underlying molecular mechanisms, as well as suggest that LicA may be a promising anti-metastatic agent.

## Introduction

Hepatocellular cell carcinoma (HCC) has been diagnosed in more than half a million people worldwide. Risk factors for the development of HCC include viral hepatitis (i.e., hepatitis B virus and hepatitis C virus), alcoholic liver disease, potentially nonalcoholic fatty liver disease, and some other rare etiologies, such as hereditary hemochromatosis, autoimmune hepatitis, and Wilson’s disease [1]. Studies have reported that the development of HCC could be caused by multiple risk factors rather than a single risk factor, and that after HCC develops, distant metastasis becomes an importance index of prognosis [2], [3]. Chemoprevention of cancer with dietary bioactive compounds may potentially reverse, suppress, or prevent cancer progression [4], [5]. In recent years, despite encouraging findings from clinical trials and studies regarding the efficacy of antiviral therapy for viral hepatitis, as well as surveillance and treatment of HCC, there are still many issues that remain unresolved, such as drug resistance toward HCC therapy and the mechanisms by which HCC metastasizes. Therefore, it is important to inhibit the spread of tumor cells to prevent the development of metastasis. Accordingly, many dietary bioactive components have shown promising anti-cancer activities with little or no toxicity to normal cells [6].

Licochalcone A (LicA) is a characteristic chalcone of licorice, which is the root of Glycyrrhiza inflate [7]. It is the most potent component of licorice and has been shown to have anti-inflammatory [8], anti-angiogenesis [9], and anti-tumor properties [10]–[12]. LicA has been shown to induce prostate cancer apoptosis via modulation of bcl-2 protein expression [13]. Additionally, LicA was shown to suppress the migration of endothelial cells and proliferation of smooth muscle, which reduced extracellular signal-regulated kinase 1/2 (ERK1/2) activity and Rb phosphorylation, thereby blocking the progression of the cell cycle [14]. Moreover, mice fed with LicA had a significant reduction in tumor formation and the number of cells expressing proliferating cell nuclear antigen, beta-catenin, cyclooxygenase-2 (COX-2), and inducible nitric oxide synthase (iNOS) in the colon, a significant increase in survival, and an inhibition of liver metastasis and expression of matrix metalloproteinase-9 (MMP-9) in the liver [11]. LicA was also found to inhibit vascular endothelial growth factor receptor 2 (VEGFR-2) signaling, which results in the inhibition of angiogenesis and tumorigenesis both *in vitro* and *in vivo* [9].

For distant metastasis to occur, malignant cells must undergo several changes. First, the cells gain the ability to migrate and invade other tissues; second, they destroy their intercellular relationships; third, the extracellular matrix (ECM) is lysed; and fourth, there is an increase in the adhesive ability between the cells and ECM [15]. Degradation of the ECM via proteolytic enzymes is a crucial step in tumor metastasis. Among these enzymes, MMPs and urokinase plasminogen activator (uPA) play a key role in degrading the ECM, allowing metastatic cells to access the vasculature, invade, and migrate into the target organ, and thereby result in tumor metastasis [16]–[17]. In addition to MMPs, the mitogen-activated protein kinase (MAPK) surperfamily members are associated with increased migration, invasion, proliferation, survival, and apoptosis, thus serving different roles in cellular responses [18]. ERK1/2, p38 MAPK, and JNK/SAPK play a central role in the regulation of MMP and uPA expression [17].

Many reports have previously shown that nuclear factor κB (NF-κB), a transcription factor, plays an important role in carcinogenesis responses. Several genes involved in tumor metastasis have also been identified as being regulated by NF-κB [19]. The promoter of MMPs and uPA is highly conserved and was shown to contain multiple functional elements, including NF-κB elements [20]–[21]. Some studies demonstrated that NF-κB promotes the migration and metastasis of HCC cells [22], cervical cancer cells [23], and breast cancer cells [24] through an upregulation of MMPs and uPA.

Little is known about the molecular mechanisms responsible for the anti-metastatic properties of LicA, and no studies have investigated the potential role of LicA in inhibiting the induction of migration and invasion of human HCC cells. Therefore, in the present study, we investigated the role of LicA in suppressing the migration and invasion of HCC cells, and the molecular mechanisms responsible for its anti-metastatic properties.

## Materials and Methods

### Reagents and Antibodies

LicA was purchased from Sigma (St. Louis, MO), and a 100 mM stock solution was prepared in dimethyl sulfoxide (DMSO) and stored at −80°C. DAPI and MTT (3-[4,5-dimethylthiazol-2-yl]-2,5-diphenyltetrazolium bromide) were purchased from Sigma (St. Louis, MO). Antibodies against p-ERK1/2, ERK1/2, p-p38, p38, p-JNK, JNK1/2, uPA, ATF-2, NF-κB (p65), c-jun, c-fos, β-actin, and si-MKK4 were purchased from Santa Cruz Biotechnology (Santa Cruz, CA). Horseradish peroxidase-conjugated anti-mouse, anti-goat and anti-rabbit secondary antibodies were obtained from Santa Cruz Biotechnology (Santa Cruz, CA). The JNK1/2 inhibitor, SP600125, was purchased from Calbiochem (San Diego, CA).

### Cell Culture

HA22T/VGH and SK-Hep-1 cells were obtained from American Type Culture Collection (ATCC, Manassas, VA, USA) and Bioresources Collection and Research Center (BCRC, Taiwan), and cultured in DMEM or MEM medium. Primary normal hepatocytes were derived from healthy rat livers and maintained in RPMI-1640 medium. Both media were supplemented with 10% fetal bovine serum (FBS; HyClone, Logan, UT, USA), 100 mM nonessential amino acid, 2 mM glutamate, 100 U/ml penicillin, and 100 µg/ml streptomycin (Cambrex, Walkersville, MD, USA). The cultures were incubated at 37°C in a humidified atmosphere with 5% CO_2_.

### Cell Viability Assay

Cell viability was determined with an MTT assay [25]. Cells were seeded at adensity of 3×10^4^ cells/well in a 24-well plate and cultured for 24 h. Cells were treated with various concentrations of LicA for 24 or 48 h. Subsequently, the medium was replaced with fresh medium containing 0.5 mg/ml MTT for 4 h. MTT is converted into formazan by dehydrogenases in the mitochondria of live cells. Thus, the number of viable cells is proportional to the amount of formazan. The medium was removed, and the produced formazan was dissolved in isopropanol and measured at 570 nm by a Multiskan MS ELISA reader (Lab systems, Helsinki, Finland). The relative number of cells was normalized to the absorbance from the untreated cells.

### Migration and Invasion Assays

A cell migration and invasion assay was performed, as previously described with slight modifications [26]. For the migration assay, experimental procedures were similar to the invasion assay described below, except the polyvinylpyrrolidone-free polycarbonate filters (Millipore; 8-mm pore size) were not coated with Matrigel (50 µg/filter). The experimental cells (2×10^5^ cells/well) were treated with LicA (0, 5, 10, and 20 µM), then trypsinized and resuspended in serum-free medium, and placed in the upper chamber of the Matrigel non-coated well insert (5×10^4^ cells/well). DMEM containing 20% FBS was placed in the lower chamber, and SK-Hep-1 and HA22T/VGH cells were then incubated for 12 h at 37°C. For the invasion assay, 10 µL of Matrigel (BD Biosciences, Bedford, MA) was applied to 8-mm pore size polycarbonate membrane filters, with the bottom chamber of the apparatus containing DMEM with 20% fetal bovine serum. SK-Hep-1 and HA22T/VGH cells were then incubated for 24 h at 37°C, and then invading cells in the membrane were fixed with methanol, stained with 0.05% Giemsa, and then counted under a light microscope at ×200. This experiment was performed twice independently. The data were presented as means ± standard deviation of triplicate samples at 5 fields.

### Detection of uPA Activity by Casein Zymography

The uPA activity was examined by casein zymography. The experimental cells (2×10^5^/wells) were plated in 6 cm dishes and treated with different concentration of LicA for 24 h. The conditioned medium was collected, separated by electrophoresis on 10% sodium dodecyl sulfate polyacrylamide gel containing 0.1% casein, and then the gels were soaked in 2.5% Triton X-100 in ddH_2_O twice for 10 min at room temperature, and incubated in substrate buffer (50 mmol/L of Tris-HCl, 5 mmol/L CaCl_2_, 0.02M NaN_3_ and 1% Triton X-100, pH 8.0) at 37°C for 18 h. Bands corresponding to uPA activity were visualized by negative staining using 0.3% Coomassie blue in 50% methanol and 10% acetic acid.

### Reverse Transcriptase Polymerase Chain Reaction (RT-PCR)

Cells were treated with the Trizol reagent (Invitrogen, Carlsbad, CA) for total RNA extraction [27]. Total RNA (2 µg) was reverse transcribed to complementary DNA (cDNA) in a reaction mixture containing 2.5 µM of oligo (dT) primers, 0.5 mM of dNTP mixture, 200 U of SuperScript III reverse transcriptase, and RNase inhibitor (Invitrogen), and incubated at 42°C for 65 min. After incubation, the reaction mixture was heat inactivated at 85°C for 5 min. PCR was performed on a reaction mixture containing 2 µL of cDNA, 0.2 mM of dNTP mixture, 2 µM of each primer, 1 U of Taq DNA polymerase, and 10× PCR Buffer (Promega). Denaturation was performed at 95°C for 5 min, followed by amplification for the indicated number of cycles at 95°C for 30 sec, 54°C for 30 sec, and 72°C for 1min 30 sec. The specific primer sequences used were as follows: uPA: 5′-TTGCGGCCATCTACAGGAG-3′ (forward), 5′-ACTGGGGATCGTTATACATC-3′ (reverse), and β-actin: 5′-GCACTCTTCCAGCCTTCCTTCC-3′ (forward), 5′-TCACCTTCACCGTTCCAGTTTTT -3′ (reverse). Each PCR product was then run on a 1.5% agarose gel and the bands were visualized under UV light. β-actin primers were used as an internal control and were equally loaded.

### Preparation of Whole-cell Lysates and Nuclear Extracts

The cells were lysed with iced-cold RIPA buffer (1% NP-40, 50 mM of Tris-HCl and 150 mM of NaCl [pH 7.5], 10 mg/mL PMSF, and 15 mg/mL sodium orthovanadate). Samples were mixed for 30 min on ice, and then centrifuged at 12,000 g for 10 min. Supernatants were then collected, denatured, and subjected to SDS-PAGE and Western blotting. Additionally, nuclear extracts from LicA-treated cells were obtained by using a Ready Prep Cytoplasmic/Nuclear Protein Extraction kit (Bio-Rad Laboratories), as per the manufacturer’s instructions [28]. Protein content was determined by using a Bio-Rad protein assay reagent with bovine serum albumin as the standard.

### Western Blotting

Western blotting was performed as previously described with slight modifications [29]. Equal amounts of protein extracts (30 µg) were separated by 10 or 12.5% SDS-PAGE and transferred onto a polyvinylidene fluoride (PVDF) membrane (Millipore, Belford, MA). After blocking, the membrane was hybridized with primary antibodies against uPA, NF-κB (p65), ATF-2, c-jun, c-fos, ERK1/2, p-ERK1/2, p38, p-p38, JNK1/2, p-JNK1/2, β-actin, Lamin B, and α-tubulin at 4^o^C overnight. After washing, the membrane was incubated with HRP-conjugated anti-mouse, anti-goat, or anti-rabbit antibody at room temperature for 2 h. Subsequently, proteins were visualized by the addition of HRP substrate with enhanced chemiluminescence, and the intensities of the bands were quantified with a Luminescent Image Analyzer LAS-4000 mini.

### Immunofluorescence Assay

Cells were seeded onto an 8 well Lab-Tek Chambered coverglass (Thermo, Rochester, NY). The next day, media were replaced with or without 20 µM LicA and cultured for 24 h. After removing the chamber, slides were rinsed with phosphate-buffered saline, and the cells were fixed with 4% paraformaldehyde and permeabilized in methanol. After washing with phosphate-buffered saline, slides were blocked with 2% bovine serum albumin. Primary and secondary antibodies were incubated in 5% bovine serum albumin. DAPI reagent was used as a mounting and counterstaining media.

### Transient Transfection

Transfections were performed with a Lipofectamine 2000 Transfection Reagent (Invitrogen). Cells seeded onto a 60-mm dish were cultured in DMEM supplemented with 10% FBS at 37°C for 24 h. Briefly, 5 µL of Lipofectamine and 3 µg of DNA were diluted in 100 µL of DMEM and allowed to equilibrate at room temperature for 5 min after mixing. The lipofectamine-DNA complex was then added to HCC cells and incubated for 6 h. Cells were then washed with PBS and replenished with DMEM containing 20% serum. After transfection, cells were incubated with 20 µM of LicA for 24 h. Cells were then lysed for Western blotting, and examined and photographed by immunofluorescence microscopy.

### uPA Promoter Activity Assay

Luciferase activity assay was performed as previously described with slight modifications [30]. Briefly, SK-Hep-1 cells were cultured in 24-well dishes, and then transiently co-transfected with 1 µg of pGL3-uPA luciferase reporter constructs and 1 µg of β-galactosidase reporter plasmid with the Lipofectamine 2000 transfection reagent, followed by various concentration of LicA for 24 h. Luciferase and β-galactosidase activities were determined with the luciferase and β-galactosidase enzyme assay system (Promega). The luminescence was measured using multilabel plate reader (Perkin Elmer). Luciferase activity was normalized to the β-galactosidase activity in cell lysates, and the mean of three independent experiments was calculated.

### Chromatin Immunoprecipitation (ChIP)

The ChIP assay was performed as previously described with slight modifications [31]. Cells were fixed with 1% formaldehyde for 10 min, and then lysed in RIAP buffer (1% NP-40, 10 mM of EDTA, and 50 mM of Tris-HCl [pH 8.0]). The lysates were sonicated to reduce DNA fragments to 200−500 base pairs. The samples were pre-cleared with protein A/G-agarose beads (40 µL) before 2 µg of NF-κB (p65) antibodies were added to the chromatin samples and incubated at 4°C overnight. Then, treated beads (40 µL) was added to the chromatin samples and incubated at 4°C for 1 h. The beads were washed three times and immunoprecipitated. DNA fragments were recovered with DNA purification kit. DNA was dissolved in 25 µL of TE buffer (10 mM of Tris, 1 mM of EDTA [pH 8.0]). Using immunoprecipitated samples, the NF-κB region in the uPA promoter was assessed with PCR. PCR primers for the uPA promoter were as follow: uPA forward, 5′-AGCATGACAGCCTCCAGCCAAGTA-3′, and uPA reverse 5′-ACGTGACCAGAACATAAACAGAGA -3′. PCR products were analyzed on 2% DNA agarose gels and images were analyzed with densitometric measurements.

### Statistical Analysis

Data were presented as mean ± standard error (S.E.) for all independent experiments performed in triplicate. Statistical differences between groups were determined with a student’s t-test by using Instat software (GraphPad Prism 4, San Diego, CA). Differences were considered to be statistically significant if the *p*-value was <0.05.

## Results

### LicA Inhibits Migration and Invasion Properties of SK-Hep-1 and HA22T/VGH Cells

**Figure 1:**
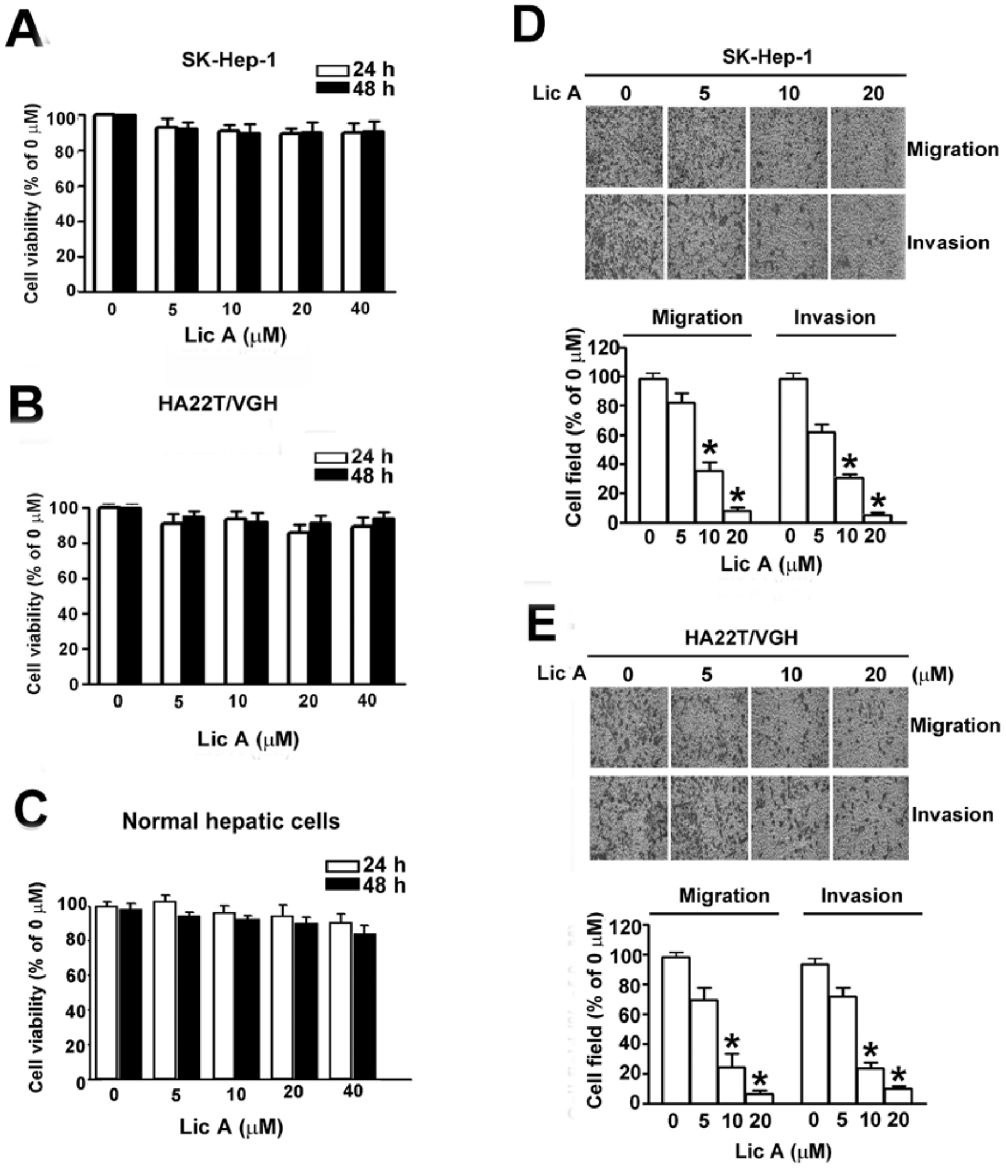
Effect of LicA on the viability, migration, and invasion of HCC cells. (A) SK-Hep-1, (B) HA22T/VGH and (C) normal hepatic cells (4×10^4^ cells/mL) were treated with various concentrations (0, 5, 10, and 20 µM) of LicA for 24 and 48 h. Cell viability was determined by using an MTT assay. (D) For the Boyden chamber migration assay, cells were treated with various concentrations of LicA for 24 h, and then cell migration was measured using a Boyden chamber for 12 h with polycarbonate filters (8 µm). (E) Cell invasion was measured using the Boyden chamber for 24 h; the polycarbonate filters were pre-coated with Matrigel. Motility was quantified by counting the number of cells that migrated to the undersides of the membrane under microscopy (200×). Data are presented as the mean±SE of at least three independent experiments. *p<0.05, compared with that of the untreated control (0 µM).

Prior to investigating the cytotoxic effects of LicA on two human HCC cells (SK-Hep-1 and HA22T/VGH) and rat normal hepatic cells with an MTT assay, we found that LicA does not have a dose- and time-dependent inhibitory effect on the growth of the human HCC cells (Fig. 1A, 1B) and normal hepatic cells (Fig. 1C). These results indicate that LicA is non-toxic to human HCC cells and normal hepatic cells at concentrations of 5∼20 µM, and accordingly, these concentrations were used for further *in vitro* experiments. Cancer cell migration is a process regulated by matrix degrading proteinases, integrins, and several other cell adhesion molecules, which can be assessed by a wound healing assay [32]. Thus, the effects of LicA on cell migration and invasion were investigated. LicA repressed cell migration in a dose-dependent manner. The migrate of cells treated with 10 and 20 µM LicA was reduced by 55.0% and 90.2%, respectively, in SK-Hep-1 cells (Fig. 1D, upper), and 62.5% and 92.4%, respectively, in HA22T/VGH cells (Fig. 1E, upper). Then, to examine the invasion of SK-Hep-1 and HA22T/VGH cells following LicA treatment, a Boyden chamber coated with Matrigel was used. Treatment with LicA decreased the invasive ability of cells in a dose-dependent manner. The invasion of cells treated with 10 and 20 µM LicA was reduced by 52.2% and 89.3%, respectively, in SK-Hep-1 cells (Fig. 1D, below), and 57.8% and 90.7%, respectively, in HA22T/VGH cells (Fig. 1E, below). These findings suggest that treatment with LicA inhibits human HCC cell migration and invasion.

### LicA Inhibits uPA Activity, Expression, and mRNA levels in SK-Hep-1 and HA22T/VGH HCC Cells

**Figure 2:**
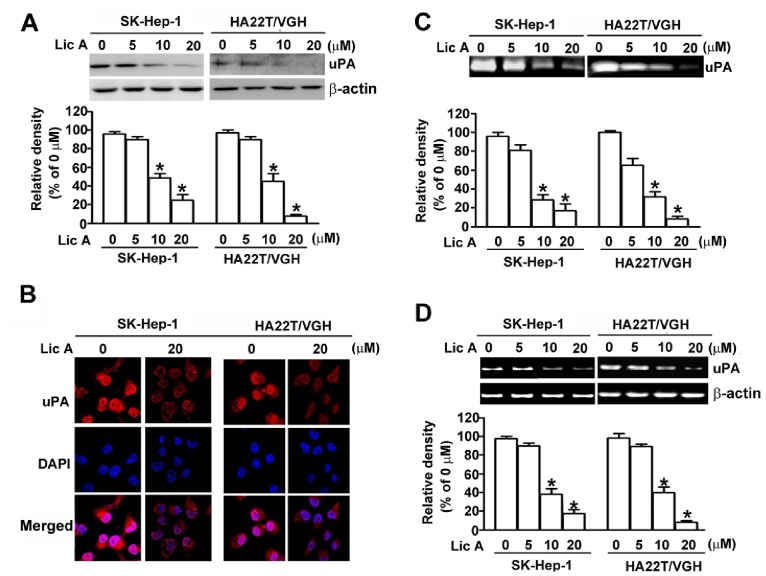
Effect of LicA on uPA expression levels in SK-Hep-1 and HA22T/VGH cells. SK-Hep-1 and HA22T/VGH cells (4×10^4^ cells/mL) were treated with various concentrations (0, 5, 10, and 20 µM) of LicA for 24 h. (A) Total protein lysates from treated cells were assessed for uPA protein levels by Western blotting, with β-actin used as a loading control. (B) Cells were fixed and immunostained with anti-uPA antibody (red), and cell nuclei were counterstained with DAPI reagent. (C) Conditioned media was collected and uPA activity was measured using casein zymography. uPA activity levels were quantified by densitometric analysis. (D) Total RNA was isolated from LicA-treated cells and subjected to RT-PCR, with β-actin as serving as an internal control. Data are presented as the mean±SE of at least three independent experiments. *p<0.05, compared with that of the untreated control (0 µM).

In addition to facilitating tumor invasion, extracellular factors, such as uPA, can modulate migration, cancer cell proliferation, and metastasis [33]. To elucidate the underlying mechanisms responsible for the anti-metastatic properties of LicA on SK-Hep-1 and HA22T/VGH cells, we detected changes in uPA expression by Western blotting, casein zymography, immunofluorescence staining, and RT-PCR. As shown in Fig. 2, Western blotting revealed that LicA treatment significantly decreased the expression of uPA in a dose-dependent manner (Fig. 2A). Treatment with 10 and 20 µM of LicA for 24 h resulted in a decrease of uPA protein levels of 55.4% and 82.3%, respectively, compared with controls in SK-Hep-1 cells, and a decrease of 74.5% and 82.6%, respectively, in HA22T/VGH cells. Furthermore, expression levels of uPA following treatment with LicA had similar dose-dependent decreases when assessed with an immunofluorescence assay (Fig. 2B). Following treatment with LicA, uPA activity levels were also assessed via casein zymography. Treatment with 10 and 20 µM of LicA for 24 h resulted in a decrease of uPA activity of 74.2% and 86.2%, respectively, compared with controls in SK-Hep-1 cells, and a decrease of 65.2% and 90.6%, respectively, in HA22T/VGH cells (Fig. 2C). Furthermore, to determine whether LicA regulated the uPA on a transcriptional level, RT-PCR analysis was performed, and it was revealed that LicA inhibited uPA mRNA levels in a dose-dependent manner (Fig. 2D). These results suggest that LicA suppressed the migration and invasion of SK-Hep-1 and HA22T/VGH cells by inhibiting uPA expression.

### LicA Inhibits the Phosphorylation of JNK in HCC Cells

**Figure 3:**
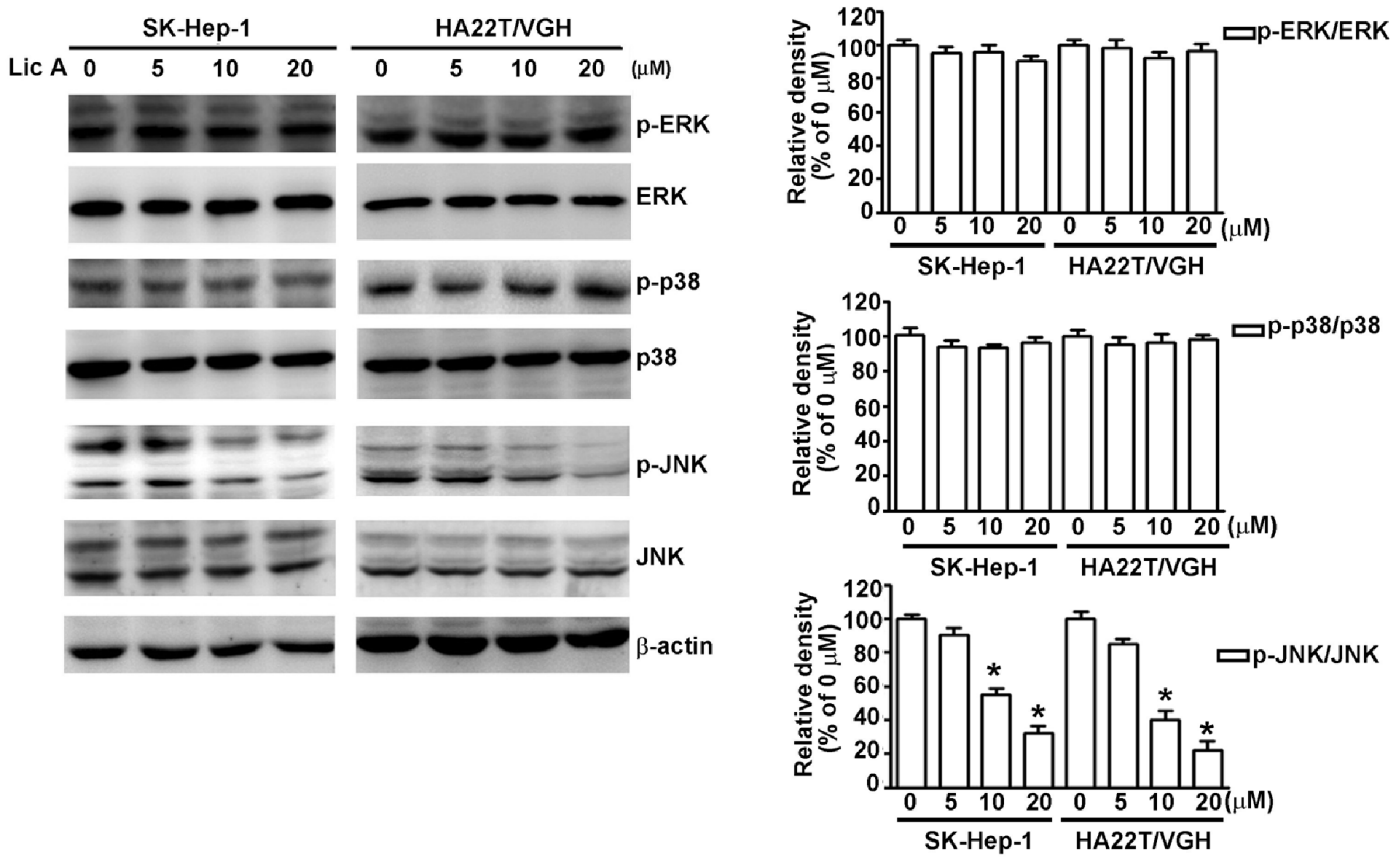
Effect of LicA on the MAPK pathway in HCC cells. HCC cells were treated with various concentrations (0, 5, 10, and 20 µM) of LicA for 24 h, and then cell lysates were subjected to Western blotting with anti-p-ERK, anti-ERK, anti-p-p38, anti-p-38, anti-p-JNK, anti-JNK, and β-actin antibodies. β-actin was used as the loading control. Data are presented as the mean±SE of at least three independent experiments. *p<0.05, compared with that of the untreated control (0 µM).

**Figure 4:**
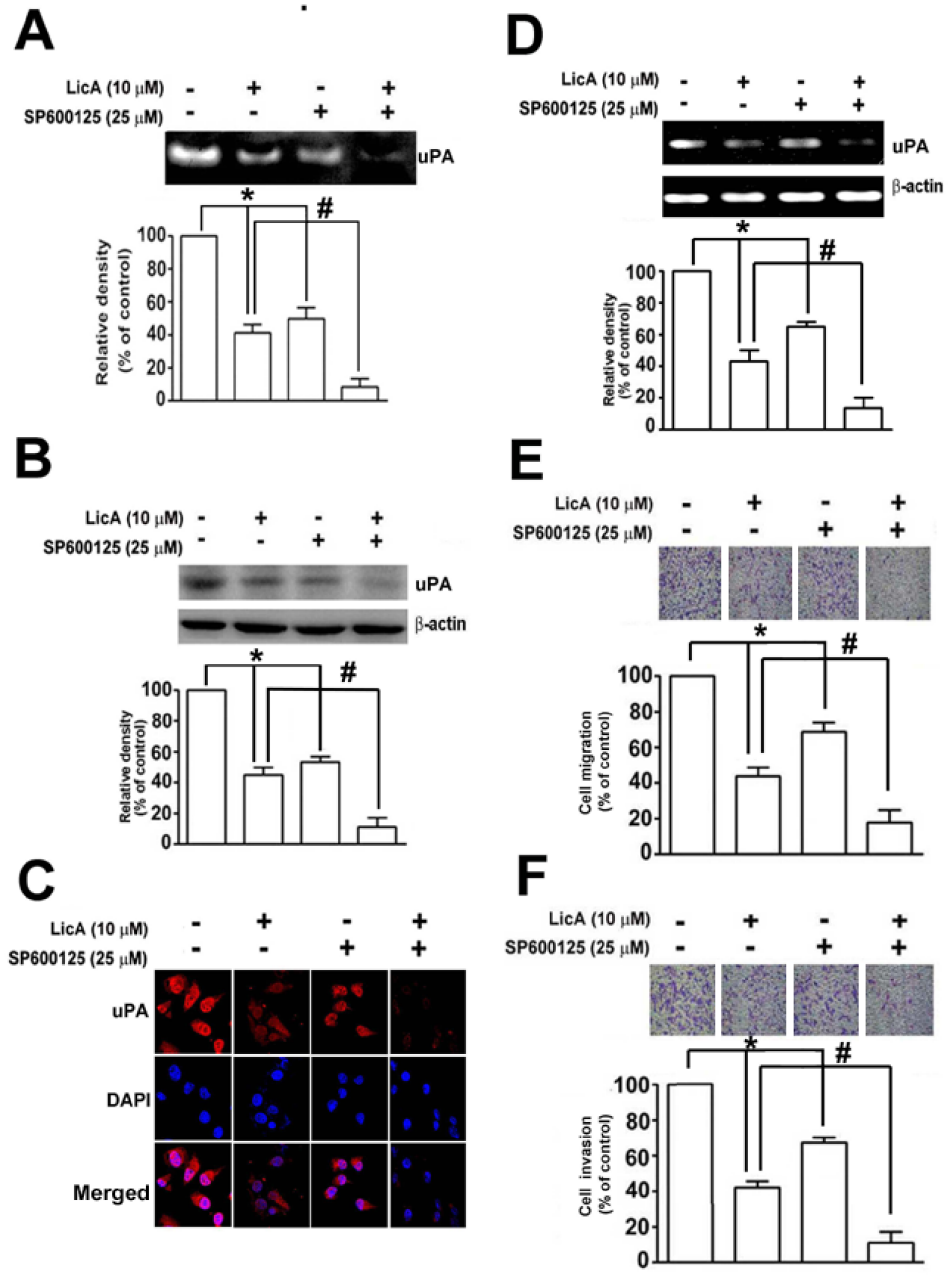
Effect of LicA on the expression of uPA, as well as cell migration and inhibition after being treated with a JNK inhibitor (SP600125). (A) SK-Hep-1 cells were pretreated with SP600125 (25 µM) for 2 h, and then incubated in the presence or absence of LicA (10 µM) for 24 h. Conditioned media was collected and uPA activity was measured using casein zymography. (B) uPA protein was determined by using western blotting. (C) Cells were fixed and immunostained with anti-uPA antibody (red), and cell nuclei were counterstained with DAPI reagent. (D) uPA mRNA expression levels were determined by RT-PCR. Cells were also assessed for migration (E) and invasion (F). Data are presented as the mean±SE of at least three independent experiments. *p<0.05, untreated cells versus SP600125 or LicA; # *p*<0.01, LicA versus SP600125 plus LicA.

MAPK has been shown to be involved in the induction of uPA in many types of cancer [34]. Given that treatment with LicA inhibits SK-Hep-1 and HA22T/VGH cell migration and invasion through an inhibition of uPA at both the transcriptional and translational level, we attempted to determine the signaling pathways involved in the LicA-induced inhibition of uPA expression in HCC cells. By treating SK-Hep-1 and HA22T/VGH cells with various concentrations of LicA, it was determined that there was a significant dose-dependent decrease in the phosphorylation of JNK1/2 in HCC cells, whereas there were no significant differences in the phosphorylation of ERK1/2 and p38 (Fig. 3). This finding indicates that LicA may suppress the expression of uPA and reduce the migration and invasion of HCC cells by inactivating the JNK1/2 pathway. To further confirm that JNK1/2 plays a role in reducing the expression of uPA, SK-Hep-1 cells were pretreated with a JNK1/2 inhibitor, SP600125 (25 µM), for 2 h and then incubated with LicA (10 µM) for 24 h. Using casein zymography, it was found that treatment with LicA (10 µM) or SP600125 (25 µM) alone reduced uPA activity by 61.2% and 51.5%, respectively. Treatment with a combination of SP600125 (25 µM) and LicA (10 µM) resulted in an 89.5% reduction of uPA activity (Fig. 4A). Using Western blotting assay, it was found that treatment with either LicA (10 µM) or SP600125 (25 µM) reduced uPA protein levels by 60.5% and 52.6%, respectively, and that combination treatment reduced uPA protein levels by 91.2% (Fig. 4B). These observations were corroborated by the results obtained with the immunofluorescence assay (Fig. 4C). Furthermore, as shown in Fig. 4D, combination treatment with LicA and SP600125 also induces a greater reduction in uPA mRNA expression (Fig. 4D). These data suggest that LicA suppresses the expression of uPA through the downregulation of JNK signaling in SK-Hep-1 cells. Moreover, in a functional assay of anti-metastatic properties, SP600125 also facilitated the LicA-induced inhibition of cell migration (Fig. 4E) and invasion (Fig. 4F).

### LicA Inhibited the Activity and Expression of uPA and Cell Migration and Invasion via the MKK4/JNK Signaling Pathway

**Figure 5:**
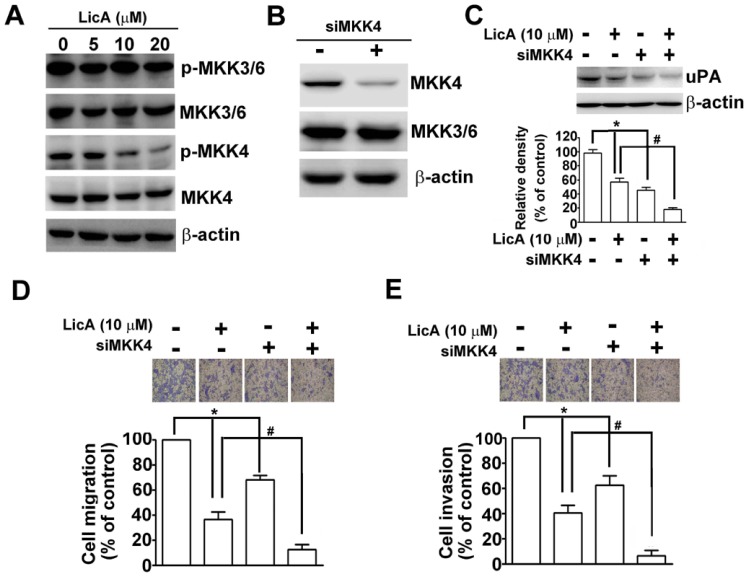
Effect of MKK4 on uPA expression levels, as well as the migration and invasion SK-Hep-1 cells. (A) Cells were treated with various concentrations (0, 5, 10, and 20 µM) of LicA for 24 h, and then cell lysates were subjected to SDS-PAGE followed by Western blotting with anti-p-MKK3/6, anti-MKK3/6, anti-p-MKK4, and anti-MKK4 antibodies. β-actin was used as an internal control. (B) Cells were treated with si-MKK4 (200 nM), and its inhibitory potency toward MKK4 or MKK3/6 was analyzed with Western blotting. (C) SK-Hep-1 cells were pretreated with si-MKK4 (200 nM) for 24 h and incubated in the presence or absence of LicA (10 µM) for another 24 h. Cell lysates were then subjected to western blotting to determine the protein expression levels of uPA. Cells were also assessed for migration (D) and invasion (E). *p<0.05, untreated cells versus si-MKK4 or LicA; # *p*<0.01, LicA versus si-MKK4 plus LicA.

MKK3/6 and MKK4 also play an important role in signaling upstream from JNK1/2 [35]. Using Western blotting, the expression levels of the phosphorylated forms of MKK4 and MKK3/MKK6 were determined after treating SK-Hep-1 cells with LicA for 24 h. As shown in Fig. 5A, LicA decreased the level of MKK4 phosphorylation, but did not affect MKK3/MKK6 phosphorylation. These data clearly suggest that LicA inhibits JNK1/2 via an MKK4-dependent pathway that is independent of MKK3/MKK6. To determine whether the inhibitory effects of LicA occur primarily via the MKK4 signaling pathway, its inhibitory specificity toward MKK3/6 and si-MKK4 was tested with a transient transfection assay. It was found that only si-MKK4 could inhibit the protein expression of MMK4 (Fig. 5B). Then, after treating experimental cells solely with either si-MKK4 or 10 µM of LicA for 24 h, it was found that there was a significant decrease in the protein expression of uPA, as assessed by Western blotting (Fig. 5C). Combination treatment with si-MKK4 and LicA induced a further reduction in uPA protein expression levels (Fig. 5C). Therefore, LicA exposure may suppress uPA expression via the MKK4/JNK signaling pathway. Accordingly, using a migration and invasion assay, we investigated whether LicA-treated cell activity was regulated by the JNK/MKK4 pathway. After being solely exposed to either si-MKK4 or LicA for 24 h, the migration and invasion of treated cells was significantly suppressed. Combination treatment with si-MKK4 and LicA induced a further inhibition of cell migration (Fig. 5D) and invasion (Fig. 5E). These data clearly demonstrate that the LicA-induced downregulation of uPA occurs via inhibition of the MKK4/JNK signaling pathway, and that this pathway plays a key role in the inhibition of cell migration and invasion.

### Inhibitory Effects of LicA on Nuclear Translocation of NF-κB and its Binding to uPA Promoter in SK-Hep-1 Cells

**Figure 6:**
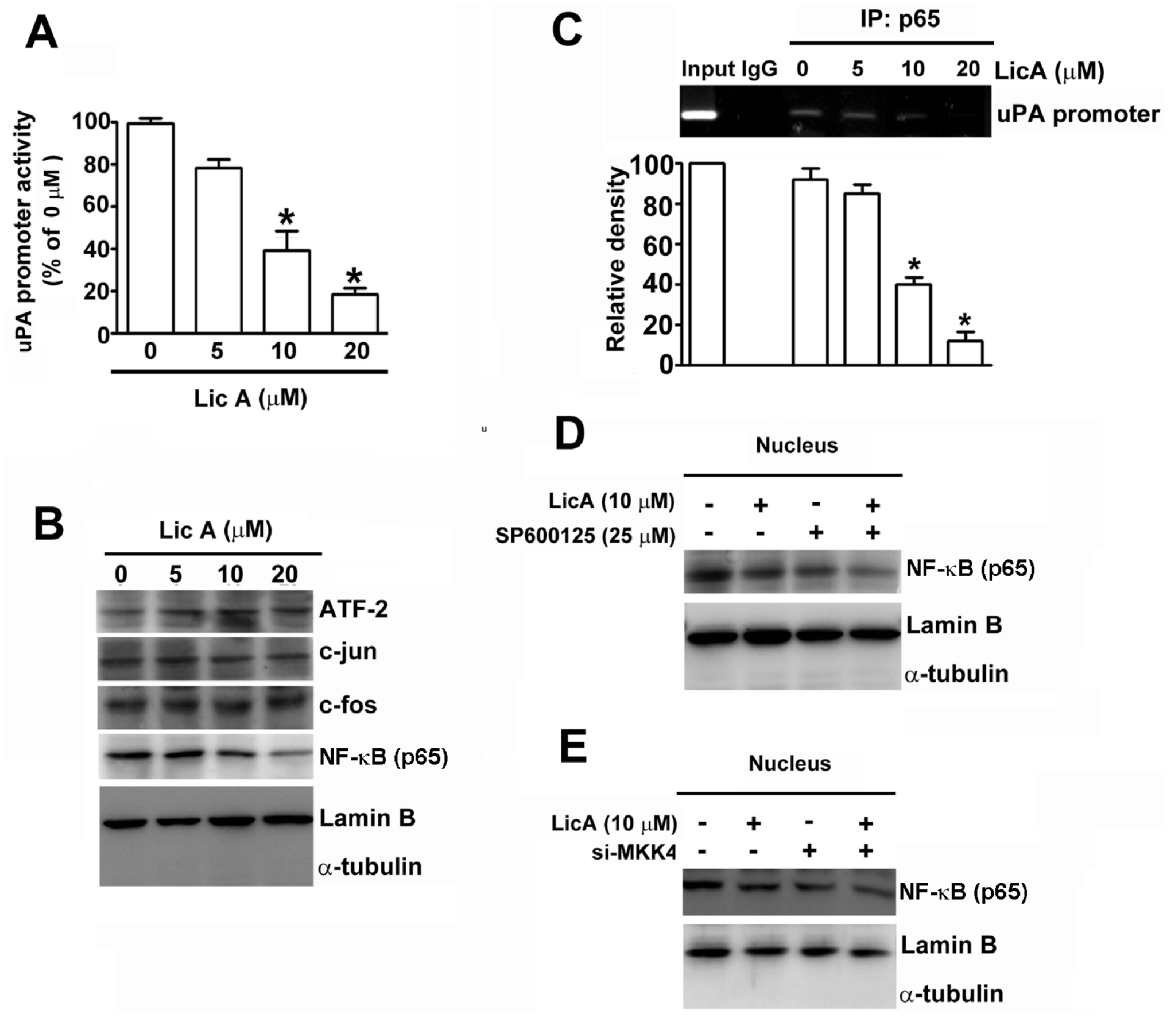
Effect of LicA on uPA transcriptional activity and NF-κB binding activity in SK-Hep-1 cells. (A) SK-Hep-1 cells transfected with a luciferase reporter plasmid containing the promoter region of uPA were treated with increased concentrations of LicA for 24 h. Cell lysates were extracted from each treatment group, and the activity of uPA promoter was determined via luciferase activity. The plot demonstrates the relative activity of the uPA promoter from three independent experiments. (B) Nuclear extracts were also assessed by Western blotting with anti-ATF-2, NF-κB (p65), c-fos, and c-jun antibodies. Lamin B and α-tubulin were used as markers of nuclear and cytosolic fractions, respectively. (C) Results from the ChIP assay on the chromatin isolated from SK-Hep-1 cells. Cells were treated with different concentration of LicA and immunoprecipitated with anti-NF-κB (p65) or control IgG. The isolates were assessed with PCR using primers targeting the sequence on the uPA promoter. The bottom plot shows the relative quantitative results compared to that of the input. (D) The nuclear localization of NF-κB after treatment with or without LicA and/or SP600125, respectively. (E) The effects of si-MKK4 and LicA on the nuclear translocation of NF-κB, cells were pretreated with si-MKK4 for 6 h and then incubated in the presence or absence of LicA (10 µM) for 18 h. Afterward, the nuclear extracts were also assessed by Western blotting to analyze the nuclear translocation of NF-κB. Bars represent the mean±S.E. from three independent experiments. *p<0.01, compared with the Input.

NF-κB, ATF-2, and AP1 are important transcription factors that translocate into the nucleus and regulate the expression of uPA secretion [36], [37]. Fig. 6A shows there was a significant concentration-dependent attenuation of uPA promoter activity in SK-Hep-1 cells treated with 0∼20 µM of LicA for 24 h. Therefore, we investigated whether LicA affects the nuclear translocation of these transcription factors and influences the transcription of uPA. The expressions of NF-κB (p65), ATF-2, c-fos, and c-jun in nuclear extracts were analyzed by Western blotting. It was found that the nuclear levels of NF-κB (p65) were significantly decreased, whereas the expression levels of ATF-2, c-fos, and c-jun were not affected (Fig. 6B). To clarify the involvement of the NF-κB transcription factor in LicA-induced inhibition of uPA transcription, ChIP assays were performed. As shown in Fig. 6C, the binding capability of NF-κB (p65) on the promoter of the uPA gene was repressed after treatment with LicA at 10 and 20 µM in SK-Hep-1 cells. To further confirm that LicA-mediated suppression of NF-κB nuclear translocation is through MKK4/JNK pathway, SP600125 was used to inactivate JNK activity, Inactivation of JNK or treatment of LicA resulted in a decrease of NF-κB nuclear translocation (Fig. 6D), and these suppressive effects could be further enhanced by combined treatment of LicA and SP600125 (Fig. 6D). In addition, SK-Hep-1 cells were transiently transfected with si-MKK4. Subsequently, the siRNA-transfected cells were exposed to the presence or absence of LicA (10 µM) for 24 h. Results have shown that a sole treatment of LicA (10 µM) or si-MKK4, respectively, reduced the nuclear translocation of the transcription factor NF-κB and that the combination treatment of LicA and si-MKK4 could repress the nuclear translocation of the NF-κB. (Fig 6E). These results suggest that LicA inhibits the nuclear translocation of the transcription factor NF-κB and reduces its binding amounts on the promoter of uPA, thereby repressing the transcription of uPA through MKK4/JNK signaling pathway in HCC cells.

## Discussion

In the present study, we found that: (1) LicA inhibits the migration and invasion of HCC cells, and does not exhibit any significant toxicity in normal hepatic cells; (2) LicA inhibits the expression and activity of uPA in HCC cells; (3) LicA inhibits the activities of MKK4 and JNK in HCC cells; and (4) LicA suppresses uPA via an inhibition of the MKK4/JNK signaling pathway, exert inhibitory effects on the nuclear translocation of the transcription factor NF-κB and its binding to uPA promoter, reduce the levels of uPA and then have an antimetastatic effect. Recent studies have reported the anti-cancer effects of LicA in various tumor cell lines, such as bladder cancer [38], gastric cancer [39], and prostate cancer cells [40]. Several studies investigating LicA have mainly focused on its anti-cancer effects or apoptotic effects in cancer cells. However, the underlying mechanisms responsible for the anti-metastatic properties of LicA in HCC cells remain largely unknown. Our study demonstrated that LicA inhibits the migration and invasion of HCC cells by downregulating MKK4/JNK/NF-κB signaling and uPA expression.

Tumor invasion and metastasis are multifactorial biological processes. Tumor cell migrate to basement membranes of cells, and eventually reach the circulatory system, which results in the development of metastatic lesions [41]. During extravasation of cancer cells that have adhered strongly to the endothelial cells of blood vessels, cancer cells degrade the basement membrane or extracellular matrix using proteases produced either by the cancer cells themselves or fibroblasts and/or other stromal cells, and then migrate through the cleavage and invade the surrounding area [42]. Hence, interrupting one or more of these steps can be an approach used in anti-metastatic therapy. In light of this, the uPA system has been reported to play a major role in extracellular matrix proteolysis and tumor invasion. When secreted uPA binds to its cell membrane-associated receptor and converts serum plasminogen to plasmin, basement membrane proteins are degraded. Studies have shown that the anti-cancer properties of several bioflavonoid-rich botanicals are associated with uPA expression. Anthocyanins have been shown to inhibit glioblastoma cell invasion through a downregulation of uPA expression [43]. Epigallocatechin-3-gallate (EGCG), a polyphenol from green tea, has been shown to suppress oral and pancreatic cancer cell invasion by inhibiting uPA expression and activity [44], [45]. LicA was reported to block colon carcinogenesis and inhibit liver metastasis, as well as suppress the expression of MMP-9 [11]. Given that, in the present study, we demonstrated that LicA downregulates uPA expression and activity, we propose that these inhibitory effects are a plausible explanation for the suppressive effects of LicA on HCC cell migration and invasion.

Induction of uPA expression involves multiple signaling cascades, particularly the MAPK pathway [46], [47]. Studies have shown that uPA activation may facilitate metastasis of human breast cancer by several mechanisms, including the Ras-ERK and p38 MAPK pathways [34]. Furthermore, green tea extract was found to modulate uPA expression via the ERK 1/2 and p38 MAPK signaling pathways in human fibrosarcoma [48], and butein was shown to inhibit uPA via the ERK, JNK, and p38 signaling pathways [47]. Isoliquiritigenin was found to inhibit the migration and invasion of DU145 cells through a suppression of JNK/AP-1 signaling [49]. Other studies have also shown that 17β-estradiol pretreatment inhibits PGE2-induced cell invasion and downregulated uPA and MMP-9 expression by suppressing JNK1/2 activation in LoVo cells [50]. Consistent with these findings, this study demonstrated that LicA inhibits the phosphorylation of JNK in conjunction with reductions in the expression and activity levels of uPA, indicating a potential underlying mechanism for LicA-induced inhibition of uPA expression.

Many studies have shown that MKK4 and its downstream mediator JNK1/2 have either oncogenic or tumor-suppressive effects depending upon the cytokine, stress signals, and probably in tumor development [51]. Recent studies suggested that MKK4 had a role in suppressing prostate and ovarian cancer metastasis. It was shown that depletion of endogenous MKK4 corresponded in increased cell motility and invasiveness. Conversely, constitutive expression of MKK4 leads to suppression of invasion through induction of the EMT [52], [53]. However, contradicting evidence indicates that high levels of MKK4 kinase expression could serve as a significant prognostic factor for relapse-free survival and for overall survival [54]. Furthermore, MKK4 has recently been shown to promote cell survival through an NF-κB-dependent pathway [55]. Our results indicate that LicA may regulate HCC cell migration and invasion by inactivating MKK4/JNK and subsequently facilitating uPA expression. Similarly, inactivation of MKK4 to reduce cell proliferation and migration through inactivation of JNK by various stimuli have been suggested in MES23.5 cells by Myricetin [56] and SK-MEL-28 cells by Isoangustone A [57]. Eupatilin inhibits PDGF-BB induced proliferation and migration in HASMC cells mediated through the attenuation of MKK4 activation [58]. LPA activates MEKK1 in a Ras-dependent manner and that dominant negative MEKK1 inhibited LPA-stimulated ovarian cancer cell migration [59].

NF-κB is constitutively activated in many cancer types, including breast cancer, and has been shown to contribute to the development and progression of tumors, including HCC cells [60]. Many studies have shown that the transcription of uPA genes is regulated by upstream regulatory sequences, including NF-κB and AP-1 binding sites [61], [62]. In one study, LicA was found to significantly inhibit TNF-α-induced DNA binding activity and the transcriptional activity of NF-κB via an inhibition of IKK activation in an inflammatory disease state [63]. LicA exerts its actions by suppressing the nuclear translocation of NF-κB and decreasing the binding of NF-κB on the uPA promoter in SK-Hep1 cells. In this respect, LicA demonstrates similar properties to that of curcumin [64], fisetin [23], and 6-shogaol/6-gingerol [65]. Herein, we also found that treatment of SK-Hep-1 cells with LicA inhibited the nuclear translocation of NF-κB, which was accompanied by an inhibition of NF-κB binding to the uPA promoter. It is thus possible that LicA may be used as an anti-metastatic agent for the treatment of HCC, as it induces a downregulation of MKK4 and JNK activation, as well as inhibits the expression of uPA, which leads to the inhibition of cell migration and invasion.

**Figure 7:**
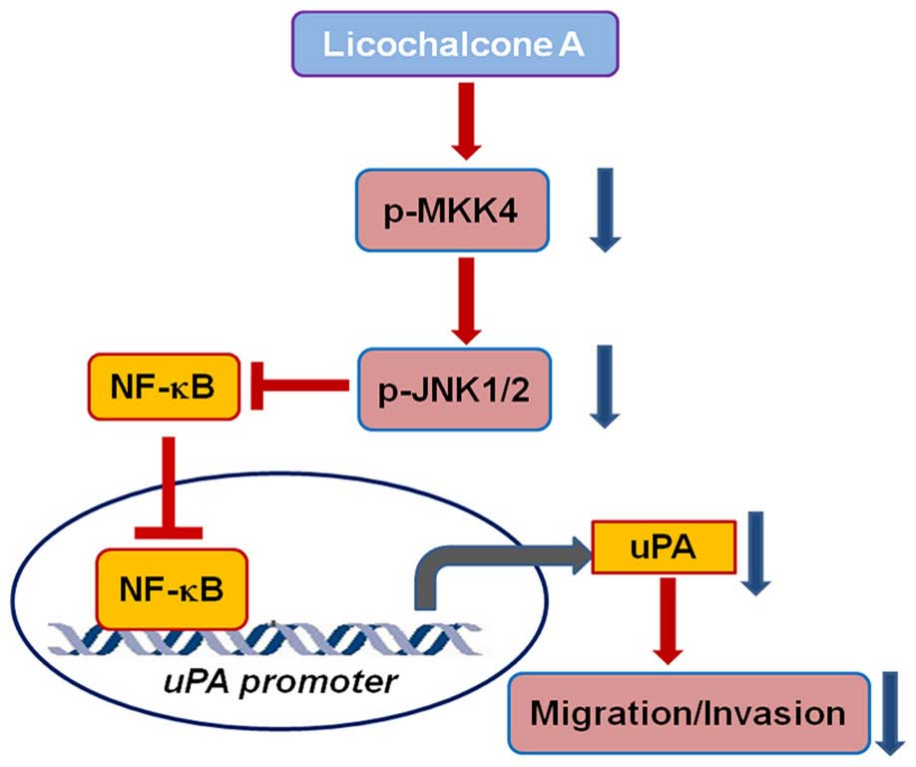
A proposed model for the molecular mechanism through which LicA inhibits HCC cell migration and invasion. LicA inhibits uPA expression and subsequent cancer cell migration and invasion through a downregulation of the MKK4/JNK signaling pathway and NF-κB transcriptional activation.

In conclusion, we demonstrated that LicA inhibits HCC cancer cell migration and invasion by decreasing uPA expression and activity. LicA-induced inhibition of uPA was found to be due to its ability to suppress the activation of the MKK4/JNK signaling pathway, as well as NF-κB nuclear translocation, and subsequently, NF-κB transcriptional activation (Fig. 7). These findings suggest that LicA may be used as an anti-metastatic agent in the treatment of human HCC.
